# Association between homocysteine and periodic limb movement during sleep in samples from the São Paulo epidemiological sleep study (EPISONO): Homocysteine and PLMS in the EPISONO study

**DOI:** 10.1007/s41105-026-00637-9

**Published:** 2026-02-23

**Authors:** Vanessa Cavalcante-Silva, Priscila K. Morelhão, Gabriel N. Pires, Vânia D’Almeida, Sergio Tufik, Monica L. Andersen

**Affiliations:** 1 Department of Psychobiology, Universidade Federal de São Paulo, Rua Napoleão de Barros, 925, São Paulo, CEP: 04024-002 Brazil; 2 Sleep Institute, Associação Fundo de Incentivo à Pesquisa, São Paulo, Brazil

**Keywords:** Sleep, PLMS, Homocysteine, Cobalamin and hemoglobin

## Abstract

Periodic limb movement in sleep (PLMS) involves repetitive, involuntary limb movements and is central to diagnosing periodic limb movement disorder (PLMD). Although distinct, PLMS frequently co-occurs with restless legs syndrome (RLS) and shares common pathophysiological factors, including deficiencies in brain dopamine, blood iron, and possibly cobalamin. Our aim was to investigate associations between blood concentrations of cobalamin, folic acid, homocysteine (Hcy), hemoglobin, iron, ferritin, and PLMS. Data from the 2007 and 2018 editions of the São Paulo Epidemiologic Sleep Study (EPISONO) were used. Elevated Hcy concentrations were identified as a risk factor for PLMS, with each 1 µmol/L increase in Hcy being associated with a 1.09-fold increase in the odds of PLMS, particularly in males. Conversely, hemoglobin concentrations were inversely associated with PLMS risk in males. Our findings suggest that Hcy may serve as a biochemical marker of PLMS risk, especially in men, while also highlighting a protective role of hemoglobin in this context.

**Supplementary Information:**

The online version contains supplementary material available at 10.1007/s41105-026-00637-9.

## Introduction

Periodic limb movement in sleep (PLMS) is a condition characterized by repetitive and involuntary limb movements, initially described as nocturnal myoclonus by Symonds in 1953 [1]. PLMS is fundamental to the diagnosis of periodic limb movement disorder (PLMD), which is defined in adults as more than 15 PLMS per hour of sleep accompanied by sleep disturbances or daytime fatigue not attributable to other causes, as outlined in the International Classification of Sleep Disorders, Third Edition (ICSD-3-TR) [2]. These limb movements occur in more than 80% of individuals diagnosed with restless legs syndrome (RLS) in Western populations [3]. In an Eastern sample, PLMS and RLS co-occurred in 57.9% of participants, decreasing to 42.3% when a PLMS index greater than 15 events per hour was used as the cutoff [4]. The phenomenon is also common among individuals without RLS symptoms, appearing in approximately 7.6–25% of the general population [5]. Its presence may impair mental, physical, social, and occupational functioning [2].

The frequent co-occurrence of PLMS and RLS [6] and the similar treatment responses for sensory symptoms support the notion of a shared underlying pathophysiology. Further evidence suggests a link between iron deficiency and the pathophysiology of these conditions [7], as studies demonstrate significant correlations between lower blood concentrations of ferritin, serum iron, and cerebrospinal fluid ferritin concentrations and increased RLS symptoms severity and periodic limb movements (PLM) frequency [8, 9]. Iron is crucial for various physiological processes in the brain, including dopamine synthesis, synaptic density maintenance, and myelin formation, and it is likely involved in norepinephrine and serotonin neurotransmitter systems as well [10]. Additionally, iron is a key component of hemoglobin as it is vital for erythroblast production, but its deficiency may be masked by a concurrent cobalamin deficiency [7]. Cobalamin deficiency impairs DNA synthesis and induces erythroblast apoptosis, reducing the number of red blood cell precursors and subsequently lowering iron demand [7]. This reduction can mask an underlying iron deficiency, resulting in deceptively normal iron concentrations despite inadequate reserves.

Cobalamin is essential for neurological health and its deficiency has been associated with various neurological disorders, including RLS [11]. Cobalamin and folic acid act as cofactors for methionine synthase in the methionine metabolism pathway, in which S-adenosylmethionine (SAM) and homocysteine (Hcy) participate as intermediates [12]. Clinical and experimental evidence supports a connection between folate, SAM, and monoamine metabolism, likely involving the biopterin pathway, an essential co-factor for tryptophan hydroxylase, the rate-limiting enzyme in the synthesis of serotonin, and also for tyrosine hydroxylase in the synthesis of dopamine [13, 14]. Dopamine deficiency is thought to contribute to the pathophysiology of both PLMS and RLS [15]. Given the role of the methionine-homocysteine pathway on monoamine metabolism, along with the potential for iron deficiency to be masked by cobalamin deficiency, the aim of the current study was to evaluate the correlation between blood concentrations of cobalamin, folic acid, Hcy, hemoglobin, iron, ferritin and the presence of PLMS in two distinct samples from the two independent EPISONO editions (2007 and 2018). Considering the impact of time on potential social and health policy changes, this approach allowed us to ensure the integrity of each cohort and conduct a robust assessment of the factors contributing to the prevalence of PLMS and their underlying mechanisms.

## Methods

### The EPISONO study – purpose and design

The São Paulo Epidemiologic Sleep Study (EPISONO) is a comprehensive population-based investigation designed to assess the prevalence of sleep disorders and related health conditions in a representative sample from São Paulo, Brazil [16]. It is performed approximately once every decade since 1987, and every new edition is based on a new representative sample, representing the city of São Paulo in terms of sex, age and socioeconomic distribution. The study protocol excluded pregnant or lactating women, individuals with severe physical or mental health conditions, and shift workers to ensure the reliability and accuracy of the findings [16].

In the 2007 edition, the EPISONO study recruited a total of 1,042 volunteers, aged 20 to 80 years, for assessments that included overnight polysomnographic (PSG) evaluations and comprehensive questionnaires covering sleep patterns and overall health. In 2018, a separate sample of 769 participants was recruited to assess the population status of the variables at two different time points. This novel sample offered further insights into the changing landscape of sleep disorders in São Paulo over time.

Ethical approval was granted by the Research Ethics Committee of the Universidade Federal de São Paulo/Hospital São Paulo (National Health Council – Resolution No. 466/2012: 0593/06, and 0985/2017).

### Data collection

In both the 2007 and 2018 editions, data collection occurred in two stages. In the first stage, trained interviewers from an independent research company (Datafolha^®^) visited randomized households. A resident from each household was selected at random and invited to participate. After reading and signing the informed consent form, each selected participant completed an initial set of questionnaires. Following the household interview, participants were invited to proceed to the second phase, held at the Sleep Institute in São Paulo, Brazil, a prominent center for sleep research. During this phase, participants completed additional sleep-focused questionnaires and underwent a full-night type 1 polysomnography (PSG), with blood samples collected the following morning for biochemical analysis. The PSG was performed respecting each participant’s usual bedtime and wake time. Participants were allowed to arrive at the sleep laboratory any time after 6 PM, and the PSG setup was scheduled at their convenience. The following morning, recordings were terminated only upon participant request, rather than at the laboratory’s standard PSG standard procedures. In both study editions, PSG assessments utilized the EMBLA^®^ S7000 digital system (Embla Systems Inc., USA) [16], with data analysis conducted in line with the American Academy of Sleep Medicine (AASM) guidelines established in 2012.

### PLMS characterization

PLMS, typically identified through polysomnography, is marked by recurrent and stereotypical limb movements during sleep. Significant PLMS is defined as having a PLM index exceeding 15 movements per hour of total sleep time [17].

### Biochemical measurements

Fasting blood samples (10–12 h) were collected and analyzed using standardized methods at the Division of Diagnostic Medicine (AFIP). The analysis included the assessment of the following categories of biomarkers: cobalamin, folic acid, Hcy, hemoglobin, iron, and ferritin. In the 2007 study phase, cobalamin, folic acid, Hcy and ferritin concentrations were measured using the acridinium ester-chemiluminescence technique, while iron and hemoglobin were quantified via the ferene–colorimetric and peroxidase methods, respectively [16]. In 2018, cobalamin, folic acid, Hcy, and ferritin were measured using the chemiluminescence method, with iron quantified using the colorimetric 2,4,6-Tripyridyl-S-triazine (TPTZ) method and hemoglobin using the cytochemical/isovolumetric method.

### Data analysis

Descriptive analyses were performed using the mean and standard deviation for continuous variables, and frequency and percentage for categorical variables. A Generalized Linear Model (GzLM) was applied to analyze the relationships between dependent and independent variables. The dependent variable was the presence or absence of PLMS, categorized using a cutoff of 15 or more PLM events per hour of sleep (indicating PLMS) or fewer than 15 PLM events per hour (indicating no PLMS). The independent variables included, cobalamin, folic acid, Hcy, hemoglobin, iron, and ferritin. There was no indication of multicollinearity as the continuous variables were not highly correlated (*r* < 0.6), and the variance inflation factor values for each variable were below 2.5 at all steps. Analyses were also stratified by sex to explore potential sex-related differences. All statistical analyses were conducted using SPSS version 29 (IBM Corp., 2023, Armonk, NY, USA).

## Results

In 2007, out of the 1,042 individuals recruited, 1,008 volunteers were included in this study, consisting of 563 women and 445 men. Thirty-four participants were excluded due to one or more missing biochemical test results. Among the remaining participants, 93 were classified as having PLMS (a prevalence of 9.2% in this sample), including 49 women and 44 men. Data on BMI, ethnicity, sleep-disordered breathing, total sleep time, sleep-related parameters, and biochemical assessments are summarized in Table 1.

Table 2 presents the results of the binary logistic regression analysis for the 2007 sample. Three separate analyses were conducted: one for the overall population, one for men, and one for women. In the analysis involving all 1,008 participants, PLMS was significantly associated with Hcy concentrations and hemoglobin. Specifically, each 1 µmol/L increase in Hcy was associated with a 1.09-fold increase in the odds of having a PLM index above 15 (95% Confidence Interval [CI] 1.04 to 1.14). Each 1 g/dL increase in hemoglobin was linked to a decreased likelihood of PLMS (Odds Ratio = 0.84; 95% CI = 0.71 to 0.97). These findings were corroborated in the male subgroup. However, in the female subgroup, these associations were no longer statistically significant.

**Table 1:** Descriptive analysis of population samples from the City of São Paulo

	All in 2007 (*n* = 1008)		Women in 2007 (*n* = 563)		Men in 2007 (*n* = 445)		All in 2018 (*n* = 744)		Women in 2018 (*n* = 435)		Men in 2018 (*n* = 309)	
	Mean	±SD	Mean	±SD	Mean	±SD	Mean	±SD	Mean	±SD	Mean	±SD
Age	42.49	14.43	43.82	14.48	40.81	14.20	48.82	14.86	50.42	14.93	46.56	14.47
IMC^a^	25.08	7.25	25.47	6.98	24.60	7.55	27.64	5.57	29.15	5.67	25.53	4.69
Total Sleep Time (minutes)	342.96	77.17	340.38	78.45	346.21	75.48	348.85	84.29	349.24	80.94	348.30	88.92
Efficiency (%)	81.94	12.87	81.66	13.40	82.29	12.17	74.58	14.93	75.36	14.24	73.43	15.80
N1 (%)	4.56	3.33	4.34	3.25	4.85	3.41	10.94	9.45	9.73	8.28	12.64	10.68
N2 (%)	54.66	9.23	54.60	9.03	54.73	9.49	47.90	9.55	47.91	9.16	47.87	10.09
N3 (%)	21.85	8.06	22.54	8.12	20.99	7.92	21.07	8.88	22.72	8.93	18.73	8.27
REM sleep (%)	18.91	6.52	18.51	6.72	19.41	6.22	20.13	7.51	19.66	7.16	20.80	7.93
Homocysteine (µmol/L)	10.00	3.71	8.99	2.65	11.28	4.41	11.69	6.45	10.92	6.94	12.78	5.53
Cobalamin (pg/mL)	512.74	240.89	520.94	232.59	502.38	250.88	413.77	173.04	420.83	185.42	403.85	153.69
Folic acid (mg/dL)	12.51	5.11	13.37	5.21	11.42	4.77	9.77	4.58	10.48	4.00	8.77	5.14
Hemoglobin (g/dL)	14.49	1.41	13.69	1.05	15.50	1.13	13.85	1.45	13.18	1.19	14.79	1.25
Iron (µg/dL)	82.50	30.83	75.44	30.42	91.44	28.99	112.52	39.85	105.27	35.53	122.73	43.28
Ferritin (ng/mL)	143.65	143.09	81.44	88.30	222.34	159.56	173.36	178.85	98.07	76.07	279.36	223.01
	N	Percent	N	Percent	N	Percent	N	Percent	N	Percent	N	Percent
PLM index < 15	915	90.8%	514	91.3%	401	90.1%	629	84.5%	375	86.2%	254	82.2%
PLM index > 15	93	9.2%	49	8.7%	44	9.9%	115	15.5%	60	13.8%	55	17.8%
Ethnicity^b^												
White	366	52.9%	206	54.8%	160	50.6%	416	57.9%	256	61.1%	160	53.5%
Black	113	16.3%	57	15.2%	56	17.7%	93	13%	45	10.7%	48	16.1%
Pardo	162	23.4%	83	22.1%	79	25%	132	18.4%	72	17.2%	60	20.1%
Yellow	19	2.7%	8	2.1%	11	3.5%	23	3.2%	12	2.9%	11	3.7%
Indigenous	14	2%	8	2.1%	6	1.9%	14	1.9%	11	2.6%	3	1%
Not mentioned	18	2.6%	14	3.7%	4	1.3%	40	5.6%	23	5.5%	17	5.7%
Severity AHI												
Without AHI	453	44.9%	284	50.4%	169	38%	473	63.6%	308	70.8%	165	53.4%
Mild AHI	199	19.7%	94	16.7%	105	23.6%	120	16.1%	58	13.3%	62	20%
Moderate AHI	100	9.9%	43	7.6%	57	12.8%	83	11.2%	38	8,7%	45	14.6%
Severe AHI	256	25.4%	142	25.2%	114	25.6%	68	9.1%	31	7,2%	37	12%

*SD* Standard deviation, *PLM* Periodic limb movement.

^a^For Body Mass Index (BMI), the 2007 subsample comprised 527 participants (women = 292; men = 235), and the 2018 subsample comprised 741 participants (women = 432; men = 309).

^b^For ethnicity, the 2007 subsample included 692 participants (women = 376; men = 316), and the 2018 subsample included 718 participants (women = 419; men = 299).

**Table 2:** Logistic regression analysis for periodic limb movement index in 2007 sample

All the participants (*n* = 1008)	Odds Ratio	95% Confidence Interval	*p*-value
Homocysteine	1.09	1.04 to 1.14	0.00
Cobalamin	1.00	0.99 to 1.001	0.72
Hemoglobin	0.84	0.71 to 0.97	0.04
Men (*n* = 445)	Odds Ratio	95% Confidence Interval	p-value
Homocysteine	1.08	1.02 to 1.15	0.00
Cobalamin	0.99	0.99 to 1.001	0.29
Hemoglobin	0.72	0.54 to 0.95	0.02
Women (*n* = 563)	Odds Ratio	95% Confidence Interval	p-value
Homocysteine	1.06	0.96 to 1.18	0.22
Cobalamin	1.001	1.00 to 1.002	0.09
Hemoglobin	0.93	0.69 to 1.24	0.62

PLM index < 15 is the reference category.

The analysis was controlled for the covariates folic acid, iron, and ferritin.

In 2018, of the initial 769 individuals recruited, 744 were included in the final analysis using the same parameters applied in 2007. Of these participants, 435 were women and 309 were men, with 115 classified as having PLMS, yielding a prevalence of 15.5% in this sample (60 women and 55 men; Table 1). Overall, no associations were identified between the independent variables and the outcome variable. However, when stratified by sex, PLMS was significantly associated with Hcy concentrations and hemoglobin in men. Specifically, each 1 µmol/L increase in Hcy was associated with a 1.07-fold increase in the odds of PLMS (95% CI: 1.01 to 1.12). In this model, each 1 g/dL increase in hemoglobin decreased the odds of PLMS, as shown in Table 3. All analyses were adjusted for folic acid, iron, and ferritin concentrations. No statistically significant associations were observed in the model for women.

**Table 3:** Logistic regression analysis for periodic limb movement index in 2018 sample

All the participants (*n* = 744)	Odds Ratio	95% Confidence Interval	*p*-value
Homocysteine	1.02	0.99 to 1.05	0.07
Cobalamin	1.00	0.99 to 1.00	0.57
Hemoglobin	0.98	0.84 to 1.14	0.82
Men (*n* = 309)	Odds Ratio	95% Confidence Interval	p-value
Homocysteine	1.07	1.01 to 1.12	0.01
Cobalamin	0.99	0.99 to 1.001	0.49
Hemoglobin	0.94	0.74 to 1.21	0.67
Women (*n* = 435)	Odds Ratio	95% Confidence Interval	p-value
Homocysteine	1.005	0.97 to 1.04	0.78
Cobalamin	1.00	0.99 to 1.002	0.88
Hemoglobin	0.93	0.74 to 1.18	0.57

PLM index < 15 is the reference category.

The analysis was controlled for the covariates folic acid, iron, and ferritin.

**Fig. 1:**
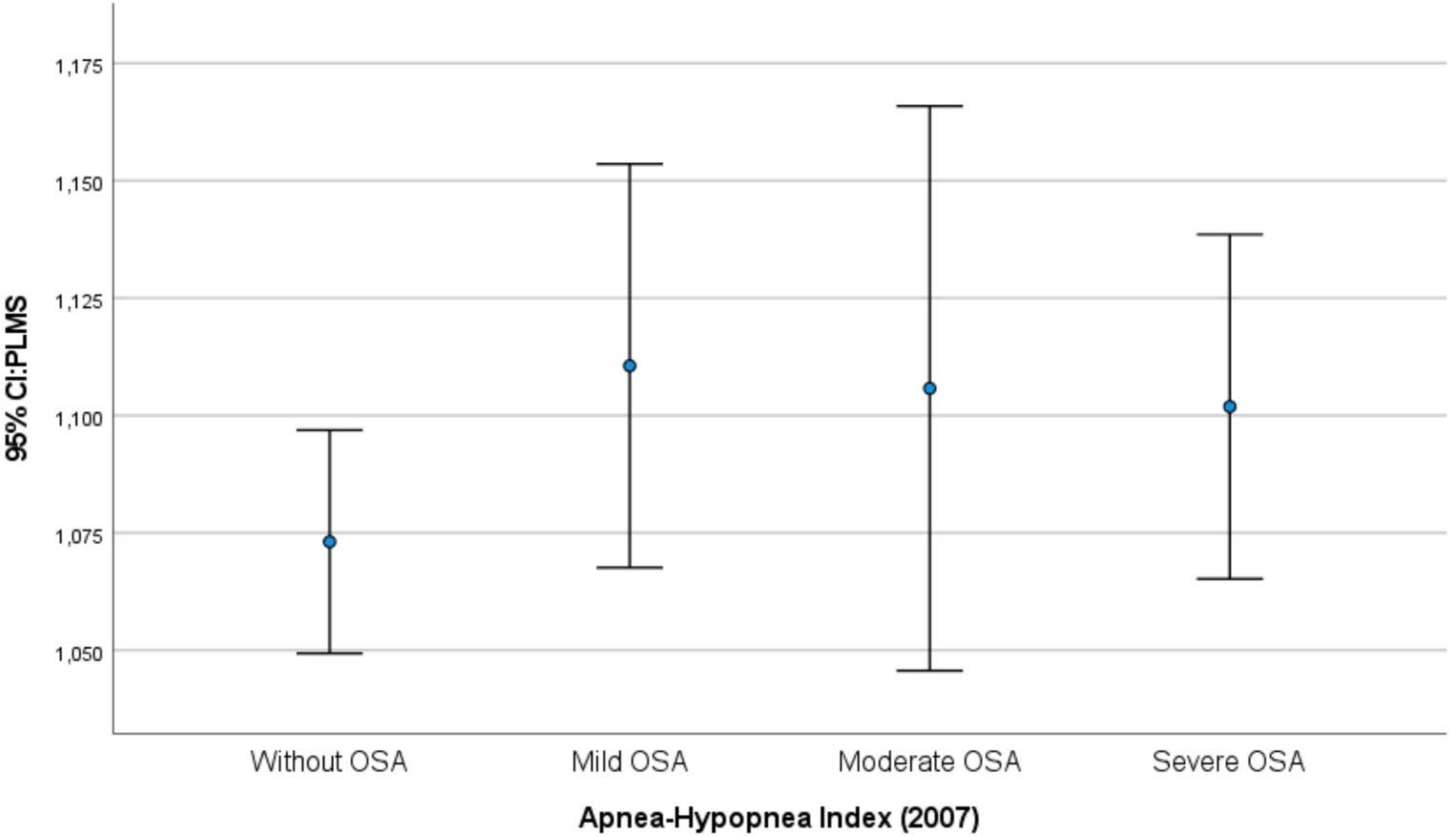
Relationship between periodic limb movement index (PLMI) and Apnea–hypopnea index (AHI) severity in the 2007 EPISONO cohort The figure illustrates the distribution of PLMI categories across AHI severity levels. A chi-square test showed no significant association between PLMI and AHI severity (χ² = 3.4, *p* = 0.33)

**Fig. 2:**
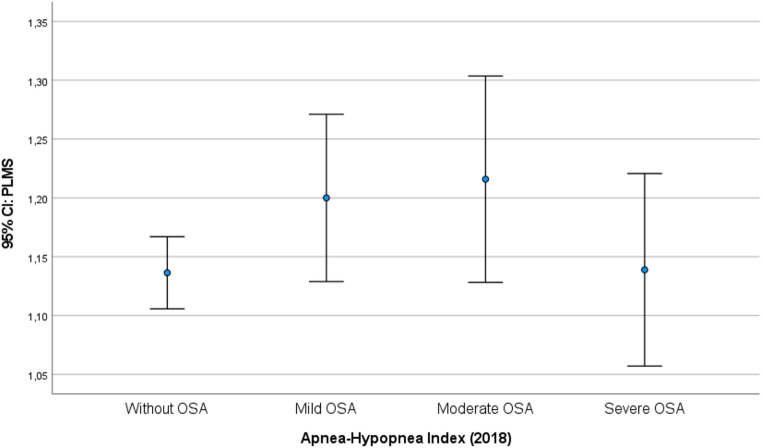
Relationship between periodic limb movement index (PLMI) and Apnea–hypopnea index (AHI) severity in the 2018 EPISONO cohort The figure depicts the distribution of PLMI categories across AHI severity groups. A chi-square test indicated no significant association between PLMI and AHI severity (χ² = 5.81, *p* = 0.12)

Figures 1 and 2 illustrate the relationship between the PLMS and apnea-hypopnea index (AHI) severity categories in the 2007 and 2018 cohorts, respectively. In both study years, the distribution of PLMS did not differ significantly across the severity levels of sleep-disordered breathing.

## Discussion

Aging, male sex, and comorbidity with RLS have consistently been identified as independent risk factors for PLMS in community-based studies [18, 19]. Our previous analyses found that although the prevalence of PLMS was higher among men, no statistically significant differences were observed between the sexes. However, we did confirm that the prevalence of PLMS increases with advancing age, and we observed that African ancestry was associated with a reduced risk of PLMS [20].

Building on these findings, the current study further extends our understanding by investigating the association between PLMS prevalence and biochemical markers, offering valuable insights into this complex relationship within the São Paulo population. Some studies have reported an association between ferritin concentrations - a blood protein that stores and releases iron - and PLMS during sleep, but the results remain inconsistent [8, 21]. Notably, low-normal ferritin concentrations (< 50 ng/mL) have been shown to correlate with severe RLS symptoms, emphasizing iron’s potential role in the pathophysiology of these sleep-related movement disorders [22]. In the current study, higher hemoglobin levels were associated with a lower risk of developing PLMS in the EPISONO 2007 sample, with each 1 g/dL increase in hemoglobin corresponding to a reduced likelihood of the condition. When the analysis was stratified by sex, this protective effect was observed only in men. However, in the EPISONO 2018 evaluation, no protective effect was found in the general population or within either sex group. Iron, as a critical component of hemoglobin, facilitates oxygen transport and supports dopaminergic function by serving as a cofactor for tyrosine hydroxylase, the enzyme responsible for dopamine synthesis [15, 23]. Iron deficiency can impair this process, resulting in reduced dopamine production and altered neurotransmission [24], especially within the basal ganglia, a brain region central to motor control. In conditions, such as RLS and PLMS, iron deficiency is often associated with disrupted dopaminergic pathways, which contribute to the characteristic symptoms of these disorders [15, 25].

Studies in both children and adults with sickle cell disease (SCD) have reported an increased rate of PLM during sleep. Mutations linked to SCD produce an abnormal beta-globin subunit, leading to the formation of Hemoglobin S. When deoxygenated, Hemoglobin S causes red blood cells to assume a rigid, sickle shape, which can obstruct the microvasculature and result in ischemia, producing the typical clinical manifestations [26]. In addition to ischemia, oxidative stress and inflammation are closely linked to the pathophysiology of SCD and its related complications [27]. Interestingly, these same features—ischemia, oxidative stress, and inflammation—are associated with elevated Hcy concentrations [28], another risk factor for PLMS identified in this study for the first time.

In the 2007 EPISONO study, a 1 µmol/L increase in Hcy was linked to a 1.09-fold increase in the odds of PLMS in the overall sample. When analyzed by sex, this elevated risk was significant only in men, corresponding to an 8% increase in risk per unit increase in Hcy. Conversely, in the 2018 EPISONO study, Hcy concentrations did not show a statistically significant association with PLMS risk in the overall sample (*p* = 0.07). However, in sex-specific analyses, each 1 µmol/L rise in Hcy was associated with a 6% increase in PLMS risk among men. Increased Hcy concentrations did not appear to be associated with cobalamin deficiency, as no significant alterations in cobalamin concentrations were observed in either the 2007 or 2018 editions of the EPISONO study. Statistical analyses were controlled for folic acid, another cofactor related to Hcy metabolism [12]. The higher Hcy concentrations observed in men are consistent with previous reports showing elevated levels across age groups, supporting the existence of intrinsic sex-related differences in homocysteine metabolism [29]. Xu and colleagues proposed a plausible biological explanation related to creatine metabolism, as men generally have greater muscle mass, leading to increased demands for creatine synthesis [30]. This process consumes methyl groups derived from S-adenosylmethionine and generates S-adenosylhomocysteine, the immediate precursor of homocysteine [12], which may partially explain the higher circulating Hcy concentrations observed in men.

The potential mechanisms linking elevated Hcy concentrations to an increased risk of PLMS may involve oxidative stress, endothelial dysfunction, and neuroinflammation [31–33] [–. Endothelial damage driven by oxidative stress and reduced nitric oxide bioavailability associated with elevated Hcy [33], may impair neuromuscular control [34] and thereby contribute to the development of PLMS. This overlap could also involve dysregulation of neurotransmitter systems. Reinforcing the possibility of shared pathophysiological mechanisms between PLMS and RLS, elevated Hcy concentrations—although with conflicting results—have been associated with RLS [11]. Previously, we found that Hcy was a predictor of increased AHI; individuals with plasma Hcy concentrations ≥ 15 µmol/L experienced an average AHI increase of 7.43 compared with those whose concentrations were < 10 µmol/L [35]. When evaluating the distribution of PLMS across the severity levels of sleep-disordered breathing, no significant differences were observed in either the 2007 or 2018 samples. Together, these findings suggest that Hcy may act as a common pathophysiological factor across different sleep disorders, potentially exacerbating conditions such as OSA and PLMS.

One of the main strengths of this study is its use of data from two EPISONO editions, conducted 11 years apart, in a large, population-based sample representative of São Paulo. The inclusion of sex-specific biochemical analyses and the investigation of novel biomarkers such as Hcy and hemoglobin significantly enriched the findings. However, this study has limitations, including the absence of a clinical evaluation to rule out other conditions related to PLMS, such as RLS, as well as the lack of an assessment for daytime impairment—both essential components for a PLMD diagnosis, which were not addressed in this research. Furthermore, when transforming cross-sectional studies into a historical series, changes in the scoring system are a possible consequence, as methodological advancements and adjustments in techniques and equipment are expected over time. However, in the current study, the analyses were performed independently, minimizing potential biases associated with these changes.

In conclusion, the findings from this study enhance our understanding of the biochemical and demographic factors associated with PLMS. Elevated Hcy concentrations were identified as a significant risk factor for the development of PLMS, potentially through mechanisms involving oxidative stress and neuroinflammation. Sex-specific biochemical alterations, particularly in men, further emphasize the need for additional research into underlying mechanisms. Additionally, hemoglobin emerged as a protective factor, possibly due to its role in supporting iron-related dopaminergic function. Considering time as a potential driver of changes in social and health policies, separately analyzing data from EPISONO 2007 and 2018 preserves the integrity of each sample and enables a robust evaluation of factors contributing to PLMS prevalence and its associated mechanisms. These insights provide a foundation for future research aimed at unraveling the mechanisms underlying PLMS and informing targeted intervention strategies.

## Supplementary Information

Below is the link to the electronic supplementary material.

Supplementary Material 1

## Appendix

See (Tables 4 and 5)

**Table 4:** Correlation matrix

Variables 2007	Homocysteine	Cobalamin	Hemoglobin	Folic Acid	Iron	Ferritin
Homocysteine	-	-0.088	0.154	-0.120	0.114	0.221
Cobalamin	-0.088	-	0.017	0.07	-0.065	0.014
Hemoglobin	0.154	0.017	-	-0.148	0.382	0.391
Folic acid	-0.120	0.070	-0.148	-	-0.05	-0.087
Iron	0.114	-0.065	0.382	-0.050	-	0.259
Ferritin	0.22	0.014	0.391	-0.087	0.259	-

Variables 2018	Homocysteine	Cobalamin	Hemoglobin	Folic Acid	Iron	Ferritin
Homocysteine	-	-0.119	0.056	-0.105	0.034	0.122
Cobalamin	-0.119	-	0.026	0.043	0.015	0.071
Hemoglobin	0.056	0.026	-	-0.106	0.394	0.339*
Folic acid	-0.105	0.043	-0.106	-	0.003	-0.120
Iron	0.034	0.015	0.394	0.003	-	0.260
Ferritin	0.122	0.071	0.339	-0.120	0.260	-

**Table 5:** Collinearity statistics

Variables 2007	Tolerance	VIF
Homocysteine	0.92	1.07
Cobalamin	0.97	1.02
Hemoglobin	0.75	1.33
Folic acid	0.95	1.04
Iron	0.84	1.18
Ferritin	0.80	1.24

Variables 2018	Homocysteine	Cobalamin
Homocysteine	0.94	1.06
Cobalamin	0.97	1.02
Hemoglobin	0.75	1.32
Folic acid	0.81	1.22
Iron	0.82	1.21
Ferritin	0.78	1.27

## References

[1] Symonds CP. Nocturnal myoclonus. J Neurol Neurosurg Psychiatry. 1953;16(3):166–71. 10.1136/jnnp.16.3.166. 13085198 10.1136/jnnp.16.3.166 PMC503132

[2] American Academy of Sleep Medicine. International classification of sleep disorders. 3rd ed. Darien: American Academy of Sleep Medicine; 2023.

[3] Montplaisir J, Boucher S, Poirier G, Lavigne G, Lapierre O, Lesperance P. Clinical, polysomnographic, and genetic characteristics of restless legs syndrome: a study of 133 patients diagnosed with new standard criteria. Mov Disord. 1997;12(1):61–5. 10.1002/mds.870120111. 8990055 10.1002/mds.870120111

[4] Shin JW, Koo YS, Lee BU, Shin WC, Lee SK, Cho YW, Jung KY. Prevalence and characteristics of periodic limb movements during sleep in Korean adult patients with restless legs syndrome. J Clin Sleep Med. 2016;12(8):1089–97. 10.5664/jcsm.6042. 27306390 10.5664/jcsm.6042 PMC4957186

[5] Gossard TR, Trotti LM, Videnovic A, St Louis EK. Restless legs syndrome: contemporary diagnosis and treatment. Neurotherapeutics. 2021;18(1):140–55. 10.1007/s13311-021-01019-4. 33880737 10.1007/s13311-021-01019-4 PMC8116476

[6] Doan TT, Koo BB, Ogilvie RP, Redline S, Lutsey PL. Restless legs syndrome and periodic limb movements during sleep in the multi-ethnic study of atherosclerosis. Sleep. 2018. 10.1093/sleep/zsy106. 29860522 10.1093/sleep/zsy106 PMC6093310

[7] Solmaz S, Ozdogu H, Boga C. Cobalamin deficiency can mask depleted body iron reserves. Indian J Hematol Blood Transfus. 2015;31(2):255–8. 10.1007/s12288-014-0417-x. 25825568 10.1007/s12288-014-0417-x PMC4375157

[8] Li J, Moore H, Lin L, Young T, Finn L, Peppard PE, et al. Association of low ferritin with PLM in the Wisconsin Sleep Cohort. Sleep Med. 2015;16(11):1413–8. 10.1016/j.sleep.2015.05.015. 26498245 10.1016/j.sleep.2015.05.015 PMC7987065

[9] Matar E, Marshall NS, Yee BJ. Efficacy of intravenous iron for restless legs syndrome-moving beyond monotherapy and into the “real world.” Sleep. 2024. 10.1093/sleep/zsae022. 38263487 10.1093/sleep/zsae022 PMC11236947

[10] Picchietti MA, Picchietti DL. Advances in pediatric restless legs syndrome: iron, genetics, diagnosis and treatment. Sleep Med. 2010;11(7):643–51. 10.1016/j.sleep.2009.11.014. 20620105 10.1016/j.sleep.2009.11.014

[11] Geng C, Yang Z, Xu P, Zhang H. Possible association between vitamin B12 deficiency and restless legs syndrome. Clin Neurol Neurosurg. 2022;223:107477. 10.1016/j.clineuro.2022.107477. 36401952 10.1016/j.clineuro.2022.107477

[12] Selhub J. Homocysteine metabolism. Annu Rev Nutr. 1999;19:217–46. 10.1146/annurev.nutr.19.1.217. 10448523 10.1146/annurev.nutr.19.1.217

[13] Bottiglieri T, Hyland K, Laundy M, Godfrey P, Carney MW, Toone BK, Reynolds EH. Folate deficiency, biopterin and monoamine metabolism in depression. Psychol Med. 1992;22(4):871–6. 10.1017/s0033291700038447. 1283223 10.1017/s0033291700038447

[14] Bottiglieri T, Laundy M, Crellin R, Toone BK, Carney MW, Reynolds EH. Homocysteine, folate, methylation, and monoamine metabolism in depression. J Neurol Neurosurg Psychiatry. 2000;69(2):228–32. 10.1136/jnnp.69.2.228. 10896698 10.1136/jnnp.69.2.228 PMC1737050

[15] Allen R. Dopamine and iron in the pathophysiology of restless legs syndrome (RLS). Sleep Med. 2004;5(4):385–91. 10.1016/j.sleep.2004.01.012. 15222997 10.1016/j.sleep.2004.01.012

[16] Santos-Silva R, Tufik S, Conway SG, Taddei JA, Bittencourt LR. Sao Paulo epidemiologic sleep study: rationale, design, sampling, and procedures. Sleep Med. 2009;10(6):679–85. 10.1016/j.sleep.2008.11.001. 19230759 10.1016/j.sleep.2008.11.001

[17] Scofield H, Roth T, Drake C. Periodic limb movements during sleep: population prevalence, clinical correlates, and racial differences. Sleep. 2008;31(9):1221–7. 18788647 PMC2542977

[18] Drakatos P, Olaithe M, Verma D, Ilic K, Cash D, Fatima Y, et al. Periodic limb movements during sleep: a narrative review. J Thorac Dis. 2021;13(11):6476–94. 10.21037/jtd-21-1353. 34992826 10.21037/jtd-21-1353 PMC8662505

[19] Leary EB, Moore H, Schneider LD, Finn LA, Peppard PE, Mignot E. Periodic limb movements in sleep: prevalence and associated sleepiness in the Wisconsin Sleep Cohort. Clin Neurophysiol. 2018;129(11):2306–14. 10.1016/j.clinph.2018.08.022. 30243181 10.1016/j.clinph.2018.08.022 PMC7750028

[20] Cavalcante-Silva V, Morelhão PK, Pires GNS, Tempaku PF, D’Almeida V, Tufik S, Andersen ML. Prevalence and incidence of periodic limb movements during sleep in São Paulo, Brazil: results from the EPISONO cohort. J Sleep Res. 2025. 10.1111/jsr.14475. 39947233 10.1111/jsr.14475

[21] Wang T, Xu J, Xu Q, Zhao R, Pan L, Zhu D, et al. Peripheral iron metabolism is associated with leg movements on polysomnography but not with the severity of restless legs syndrome or its impact on patients. Nat Sci Sleep. 2022;14:1829–42. 10.2147/NSS.S378970. 36263372 10.2147/NSS.S378970 PMC9575586

[22] Sun ER, Chen CA, Ho G, Earley CJ, Allen RP. Iron and the restless legs syndrome. Sleep. 1998;21(4):371–7. 9646381

[23] Kulaszynska M, Kwiatkowski S, Skonieczna-Zydecka K. The iron metabolism with a specific focus on the functioning of the nervous system. Biomedicines. 2024. 10.3390/biomedicines12030595. 38540208 10.3390/biomedicines12030595 PMC10968467

[24] Pino JMV, da Luz MHM, Antunes HKM, Giampa SQC, Martins VR, Lee KS. Iron-restricted diet affects brain ferritin levels, dopamine metabolism and cellular prion protein in a region-specific manner. Front Mol Neurosci. 2017;10:145. 10.3389/fnmol.2017.00145. 28567002 10.3389/fnmol.2017.00145 PMC5434142

[25] Khachatryan SG, Ferri R, Fulda S, Garcia-Borreguero D, Manconi M, Muntean ML, et al. Restless legs syndrome: over 50 years of European contribution. J Sleep Res. 2022;31(4):e13632. 10.1111/jsr.13632. 35808955 10.1111/jsr.13632 PMC9542244

[26] Patel RA, Hall DA, Eichenseer S, Bailey M. Movement disorders and hematologic diseases. Mov Disord Clin Pract. 2021;8(2):193–207. 10.1002/mdc3.13129. 33553488 10.1002/mdc3.13129 PMC7853188

[27] Antwi-Boasiako C, Dankwah GB, Aryee R, Hayfron-Benjamin C, Donkor ES, Campbell AD. Oxidative profile of patients with Sickle Cell Disease. Med Sci. 2019. 10.3390/medsci7020017. 10.3390/medsci7020017 PMC6410293 30691006

[28] Papatheodorou L, Weiss N. Vascular oxidant stress and inflammation in hyperhomocysteinemia. Antioxid Redox Signal. 2007;9(11):1941–58. 10.1089/ars.2007.1750. 17822365 10.1089/ars.2007.1750

[29] Xu R, Huang F, Wang Y, Liu Q, Lv Y, Zhang Q. Gender- and age-related differences in homocysteine concentration: a cross-sectional study of the general population of China. Sci Rep. 2020;10(1):17401. 10.1038/s41598-020-74596-7. 33060744 10.1038/s41598-020-74596-7 PMC7566483

[30] Bolanowski M, Nilsson BE. Assessment of human body composition using dual-energy x-ray absorptiometry and bioelectrical impedance analysis. Med Sci Monit. 2001;7(5):1029–33. 11535954

[31] Lai WK, Kan MY. Homocysteine-induced endothelial dysfunction. Ann Nutr Metab. 2015;67(1):1–12. 10.1159/000437098. 26201664 10.1159/000437098

[32] Moretti R, Giuffre M, Caruso P, Gazzin S, Tiribelli C. Homocysteine in neurology: a possible contributing factor to small vessel disease. Int J Mol Sci. 2021. 10.3390/ijms22042051. 33669577 10.3390/ijms22042051 PMC7922986

[33] Elsherbiny NM, Sharma I, Kira D, Alhusban S, Samra YA, Jadeja R, et al. Homocysteine induces inflammation in retina and brain. Biomolecules. 2020. 10.3390/biom10030393. 32138265 10.3390/biom10030393 PMC7175372

[34] Bukharaeva E, Shakirzyanova A, Khuzakhmetova V, Sitdikova G, Giniatullin R. Homocysteine aggravates ROS-induced depression of transmitter release from motor nerve terminals: potential mechanism of peripheral impairment in motor neuron diseases associated with hyperhomocysteinemia. Front Cell Neurosci. 2015;9:391. 10.3389/fncel.2015.00391. 26500495 10.3389/fncel.2015.00391 PMC4594498

[35] Cavalcante-Silva V, Morelhao PK, Fernandes GL, D’Almeida V, Tufik S, Andersen ML. Homocysteine as a predictor of apnea-hypopnea index in obstructive sleep apnea: a longitudinal epidemiological study (EPISONO). Eur Arch Otorhinolaryngol. 2024;281(6):3237–43. 10.1007/s00405-024-08614-z. 38568296 10.1007/s00405-024-08614-z

