# Supplementary material for: Association between homocysteine and periodic limb movement during sleep in samples from the São Paulo epidemiological sleep study (EPISONO): Homocysteine and PLMS in the EPISONO study
Source: Sleep Biol Rhythms. 2026 Feb 23;24(3):333–41. doi: 10.1007/s41105-026-00637-9 (PMC13305063; doi:10.1007/s41105-026-00637-9)
Supplement: Supplementary file 1 — Supplementary Material 1 [file 41105_2026_637_MOESM1_ESM.docx]

**Appendix**

| Table 1. Correlation matrix. | | | | | | |
| --- | --- | --- | --- | --- | --- | --- |
| Variables 2007 | **Homocysteine** | **Cobalamin** | **Hemoglobin** | **Folic Acid** | **Iron** | **Ferritin** |
| Homocysteine | - | -0.088 | 0.154 | -0.120 | 0.114 | 0.221 |
| Cobalamin | -0.088 | - | 0.017 | 0.07 | -0.065 | 0.014 |
| Hemoglobin | 0.154 | 0.017 | - | -0.148 | 0.382 | 0.391 |
| Folic acid | -0.120 | 0.070 | -0.148 | - | -0.05 | -0.087 |
| Iron | 0.114 | -0.065 | 0.382 | -0.050 | - | 0.259 |
| Ferritin | 0.22 | 0.014 | 0.391 | -0.087 | 0.259 | - |
| Variables 2018 | **Homocysteine** | **Cobalamin** | **Hemoglobin** | **Folic Acid** | **Iron** | **Ferritin** |
| Homocysteine | - | -0.119 | 0.056 | -0.105 | 0.034 | 0.122 |
| Cobalamin | -0.119 | - | 0.026 | 0.043 | 0.015 | 0.071 |
| Hemoglobin | 0.056 | 0.026 | - | -0.106 | 0.394 | 0.339* |
| Folic acid | -0.105 | 0.043 | -0.106 | - | 0.003 | -0.120 |
| Iron | 0.034 | 0.015 | 0.394 | 0.003 | - | 0.260 |
| Ferritin | 0.122 | 0.071 | 0.339 | -0.120 | 0.260 | - |

| Table 2. Collinearity statistics. | | |
| --- | --- | --- |
| Variables 2007 | **Tolerance** | **VIF** |
| Homocysteine | 0.92 | 1.07 |
| Cobalamin | 0.97 | 1.02 |
| Hemoglobin | 0.75 | 1.33 |
| Folic acid | 0.95 | 1.04 |
| Iron | 0.84 | 1.18 |
| Ferritin | 0.80 | 1.24 |
| Variables 2018 | **Homocysteine** | **Cobalamin** |
| Homocysteine | 0.94 | 1.06 |
| Cobalamin | 0.97 | 1.02 |
| Hemoglobin | 0.75 | 1.32 |
| Folic acid | 0.81 | 1.22 |
| Iron | 0.82 | 1.21 |
| Ferritin | 0.78 | 1.27 |
